# Mucin-Poor Mucinous Tubular and Spindle Cell Carcinoma of the Kidney Presented with Multiple Metastases Two Years after Nephrectomy: An Atypical Behaviour of a Rare, Indolent Tumour

**DOI:** 10.1155/2017/6597592

**Published:** 2017-11-14

**Authors:** I. Sokolakis, C. Kalogirou, L. Frey, M. Oelschläger, M. Krebs, H. Riedmiller, H. Kübler, D. Vergho

**Affiliations:** ^1^Department of Urology and Paediatric Urology, Julius Maximilian University of Würzburg, Würzburg, Germany; ^2^Institute of Pathology, Julius Maximilian University of Würzburg, Würzburg, Germany

## Abstract

**Background:**

Mucinous tubular and spindle cell carcinoma (MTSCC) is a rare type of renal cell carcinoma, whose clinical behaviour and metastatic potential have not been fully elucidated to date. There are only a few metastatic cases in the literature, which all either featured sarcomatoid differentiation or were synchronously metastasised at diagnosis.

**Case Presentation:**

We report a case of a 49-year-old male with end-stage kidney disease on dialysis, presenting with multiple osseous metastases of a mucin-poor variant of MTSCC of the kidney, without sarcomatoid differentiation, two years after bilateral nephrectomy for papillary renal cell carcinoma (RCC) at a curable stage. After retrospectively reexamining the initial nephrectomy specimens, the tumour of the right kidney was also diagnosed as a mucin-poor variant of MTSCC, while the tumour of the left kidney was confirmed as a papillary RCC.

**Conclusions:**

It is proposed that MTSCC can be associated with end-stage renal disease and that particularly the mucin-poor variant is easily confused with papillary renal cell carcinoma, as happened in this case. Although it is considered as a relatively indolent malign entity, it can metastasise even years after successful primary surgical treatment. This implies, besides accurate diagnosis, that MTSCC patients should be monitored closely in the follow-up period.

## 1. Background

Mucinous tubular and spindle cell carcinoma (MTSCC) is a rare subtype of renal cell carcinoma (RCC), which was first recognized as a distinct entity in the 2004 World Health Organization (WHO) tumour classification as well as in the newly revised 4th edition published in 2016 [1]. It is characterised by small, elongated tubules lined by cuboidal cells and/or cords of spindled cells separated by pale mucinous stroma [2]. Although it is described in the literature as a low-grade, relatively indolent tumour, it has a broad histological spectrum ranging from low to high grade tumours including sarcomatoid differentiation, which can contribute to an aggressive clinical course. A “mucin-poor” pattern of MTSCC has also been described where there is little or no extracellular mucin to be found [3]. Because of its rarity (around 100 cases reported in the literature), the clinical behaviour and metastatic potential of MTSCC remain unclear. There are only a few metastatic cases of MTSCC, which all either featured sarcomatoid differentiation or were synchronously metastasised [4]. Therefore, to contribute to the further reconditioning of MTSCC, we present a case of mucin-poor mucinous tubular and spindle cell carcinoma of the kidney presenting with metastases two years after bilateral nephrectomy.

## 2. Case Presentation

A 49-year-old male was presented at our department with lower back pain and walking instability for the last few days. The patient suffered from end-stage renal disease on dialysis for the last 15 years and had a history of successful renal transplantation about 25 years ago. The initial renal insufficiency resulted from a chronic glomerulonephritis and the allograft was rejected 9 years after transplantation due to the same underlying condition. Furthermore, about 2 years ago, he underwent bilateral nephrectomy for solid tumorous masses in both nonfunctioning kidneys. The histologic examination revealed, bilaterally at that time, type 1 papillary renal cell carcinoma (pT1aN0M0, Fuhrman grade II, in sano resection). Additionally, the nonfunctioning transplant kidney was removed about 6 months ago due to recurrent episodes of macrohaematuria, without evidence for a malign tumour. At that time, there were no signs for a metastatic disease.

**Figure 1:**
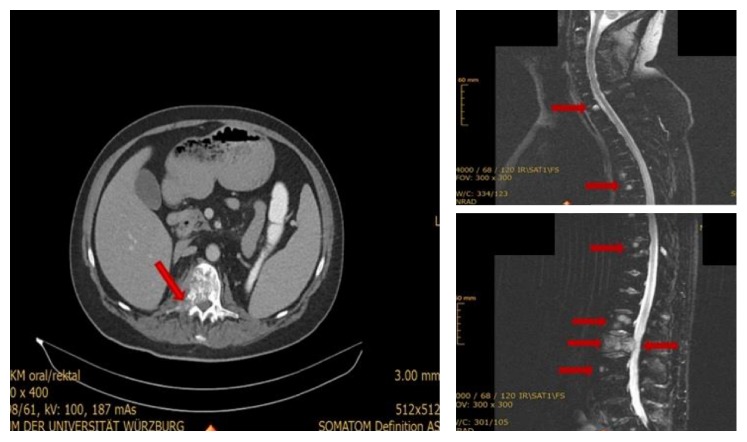
MRI and CT findings at diagnosis showing a pathologic facture at the right side of the first and second lumbar vertebral body from a metastatic solid mass, as well as multiple osteolytic metastases from 7th cervical to 2nd lumbar vertebra (arrows).

**Figure 2:**
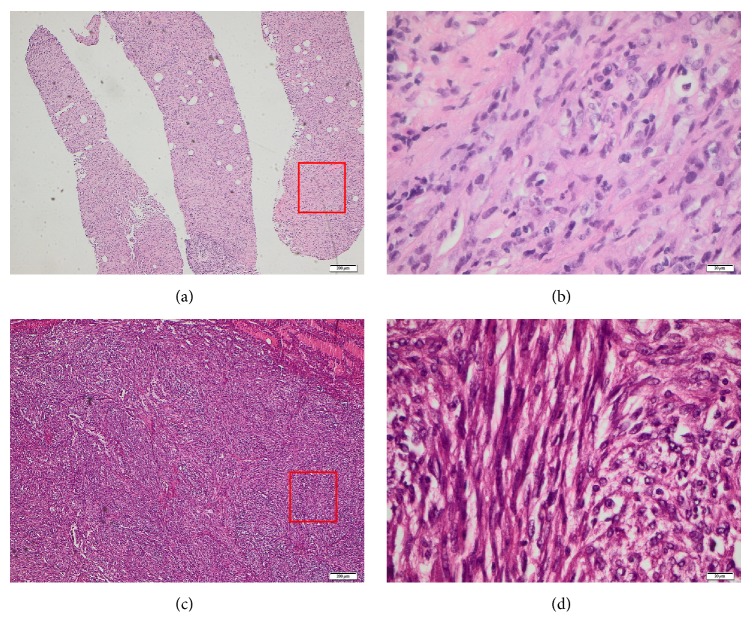
Histologic photos of the mucin-poor MTSCC from the biopsy of the metastatic lesion (a and b) and from the initial right kidney tumour specimen (c and d) showing a renal cell tumour with spindle cell component, as well as tubular and papillary parts without any clear cells.

At current presentation, the performed magnetic resonance imaging (MRI) of the spine showed a pathologic facture at the right side of the first and second lumbar vertebral body (Figure 1) from a metastatic solid mass, as well as multiple osteolytic metastases from 7th cervical to 2nd lumbar vertebra. A staging computed tomography (CT) of the thorax and abdomen showed further osseous osteolytic metastatic lesions at the sternum, both scapulae, the bone pelvis, and the left proximal femoral bone. The performed biopsy of the osteolytic metastatic lesion at the second lumbar vertebra revealed a metastatic, mucin-poor variant, of a mucinous tubular and spindle cell carcinoma of the kidney (Figure 2). A thorough reexamination of the old nephrectomy specimens showed that the tumour of the right kidney was also a mucin-poor variant of a MTSCC of the kidney, while the tumour of the left kidney was confirmed as a papillary RCC.

Histologically, both the biopsy sample and the nephrectomy-specimen of the right kidney showed a renal cell tumour with a spindle cell component, as well as tubular and papillary parts without any clear cells. Both spindle and tubular-papillary parts were delimited by a basal membrane, forming a ball-shaped, epithelial tumour-growth pattern with spindled nuclear morphology, which did not match a sarcomatoid differentiation. Immunohistochemically, the tumour was negative for desmin and actin and S100 protein, as well as for HMB45, MITF1, MART1, and SOX-10. A positive reaction to Racemase (AMARC), PAX-8, and RCC was found, with focal positivity for CD10. Pan-Cytokeratin (AE1/AE3) and CK7 were negative except from the papillary parts. The Ki67 stain showed focal proliferation rates up to 30%. Furthermore, no mucoid interstitial matrix was found with H&E stain. Only with Alcian blue staining, <1% mucin in cellular areas was observed.

According to our interdisciplinary tumour conference board's recommendation, the patient followed a systemic treatment with pazopanib and focal radiotherapy to lumbar and cervical vertebrae metastases. In further follow-up, irrespective of the started treatment, the tumour spread rapidly and after three months the patient presented with additional hepatic and pulmonary metastases. The patient finally succumbed to the disease a few months later.

## 3. Discussion

Mucinous tubular and spindle cell carcinoma (MTSCC) of the kidney shows variants raging from the “classical” type to the mucin-poor variant as described in this report. Additionally, spindle or tubular predominance and low to high grades including sarcomatoid dedifferentiation can be observed with impact on the aggressiveness of the clinical course [2, 3]. When the mucin-poor variant occurs, histologically, MTSCC can be easily confused with sarcomatoid RCC (spindle cells) or with papillary RCC, as both entities share overlapping morphological features: especially type 1 papillary RCC, which focally adopts a solid growth pattern with elongated tubules and papillae, imparting a fusiform architecture mimicking MTSCC [3]. The immunohistochemical analysis in distinguishing papillary RCC from MTSCC is also mostly unhelpful as they can share common profiles like PAX-8. The Alcian blue staining usually can reveal some mucin in cellular areas. Furthermore, MTSCC lacks the gains of chromosomes 7 and 17 and losses of chromosome Y that are typical for papillary RCC, so fluorescence in situ hybridization analysis for these chromosomes could be helpful in differentiating these two entities [3, 4]. Although a close relationship to papillary RCC has been suggested, on the basis of clinical, morphological, and molecular genetic data, MTSCC is to be considered as a separate and distinct renal neoplasm [1, 3].

Our case did not feature any sarcomatoid differentiation but presented with metastatic disease two years after successful surgical treatment (nephrectomy) at a curable stage (TNM stage: pT1aN0M0, G2, R0). Regarding this, it is therefore recommended that, although an innocent outcome is likely, a close follow-up is absolutely warranted. Furthermore, the tumour did not respond to systemic treatment with tyrosine-kinase inhibitor pazopanib, showing tumour progression with additional hepatic and pulmonary metastases only three months later. Until now, there is no guideline recommendation on systemic treatment of MTSCC due to its sporadic appearance and mostly indolent course, although recently, a case report described a response to sunitinib [5].

The underlying mechanisms of an association of MTSCC with end-stage renal disease (ESRD) are unknown. A small case series investigating RCC in patients with ESRD and the relationship between histological type and duration of dialysis showed that, in patients with >10 year on dialysis, MTSCC was relatively common, presenting in 3 out of 12 patients [6]. These results match our case, as the patient presented here was on dialysis for more than 10 years at the time of the initial diagnosis. Furthermore, in the case presented here, another contribution to the carcinogenesis of MTSCC could have been the history of immunosuppression due to the renal transplantation. For this hypothesis and MTSCC, we did not find any data in the current literature, although carcinogenesis can generally be promoted through immunosuppression [7]. Therefore, our data implies that further investigation on the relationship between ESRD, immunosuppression, and MTSCC is needed.

## 4. Conclusion

Although MTSCC is considered a relatively indolent tumour, it can metastasise even years after successful primary surgical treatment and it may be associated with end-stage renal disease. The mucin-poor variant can easily be confused with papillary renal cell carcinoma and therefore its differential diagnosis can be challenging. This implies, besides accurate diagnosis, that MTSCC patients should be monitored closely in the follow-up period.

## Abbreviations

CT: Computed tomography
ESRD: End-stage renal disease
MRI: Magnetic resonance imaging
MTSCC: Mucinous tubular and spindle cell carcinoma
RCC: Renal cell carcinoma
WHO: World Health Organization.

## Additional Points

*Availability of Data*. The datasets used and/or analysed during the current study are available from the corresponding author on reasonable request.
